# New Disturbing Trend in Antimicrobial Resistance of Gram-Negative Pathogens

**DOI:** 10.1371/journal.ppat.1000221

**Published:** 2009-03-27

**Authors:** Jung Hun Lee, Seok Hoon Jeong, Sun-Shin Cha, Sang Hee Lee

**Affiliations:** 1 Drug Resistance Proteomics Laboratory, Center for Antibiotic Resistance, Department of Biological Sciences, Myongji University, Yongin, Gyeonggido, Republic of Korea; 2 Department of Laboratory Medicine and Research Institute of Bacterial Resistance, Yonsei University College of Medicine, Republic of Korea; 3 Structural Biology Laboratory, Marine Biotechnology Center, Korea Ocean Research and Development Institute, Ansan, Republic of Korea; The Scripps Research Institute, United States of America

Gram-negative bacteria-producing extended-spectrum β-lactamases (ESBLs) are found to be truly multiresistant pathogens causing severe clinical problems. In our investigations, fifteen class C β-lactamases with extended substrate spectra have been reported in Gram-negative pathogens. Because of the emergence and dissemination of these enzymes, we propose that these enzymes be recognized as class C ESBLs (cESBLs), although most of the known ESBLs are class A and D β-lactamases. To decrease the selective pressure of antimicrobial drugs and minimize antimicrobial resistance, it is necessary for health-care professionals to recognize the presence of emerging cESBLs as a new and disturbing trend in antimicrobial resistance of Gram-negative pathogens. Because there is currently no drug development against cESBL-producing Gram-negative pathogens in progress and large pharmaceutical companies have largely withdrawn from research and development of new antimicrobial drugs, there is a tremendous need for the development of new β-lactams (or β-lactamase inhibitors) by focused cooperation between academia and small pharmaceutical companies, using the similar structural mechanism (a potential therapeutic target) of the extended substrate spectrum shown in most cESBLs.

The consensus view about antimicrobial resistance is that severe clinical problems arise from the emergence of antibiotic resistance in Gram-negative pathogens causing nosocomial infections, and from the lack of new antimicrobial agents to challenge the threat [1]. There are four disturbing trends (extending substrate spectra) in the increasing antimicrobial resistance of Gram-negative pathogens [1]: (i) class B β-lactamases (metallo-β-lactamases) conferring resistance to almost all β-lactam antibiotics [2]; (ii) a bifunctional aminoglycoside-modifying enzyme [3]; (iii) the evolution of a fluoroquinolone-modifying enzyme from an aminoglycoside acetyltransferase [4]; and (iv) a new plasmid-borne fluoroquinolone efflux determinant [5]. These disturbing trends indicate that options for the treatment of health-care–associated Gram-negative infections are perilously limited as the organisms expand their ability to evade existing antimicrobial agents [1],[6]. Here we wish to draw attention to a new disturbing trend (the recently emerging class C extended-spectrum β-lactamases [ESBLs]), and to the antimicrobial drug development for class C ESBLs. We suggest also that the category of ESBLs has to be expanded.

## Epidemiology and Characteristics of Class C ESBLs

Generally, ESBLs are defined as β-lactamases able to hydrolyze the penicillins, cephalosporins (first-, second-, and third-generation), and monobactams (aztreonam), but not the cephamycins or carbapenems [7]. In other words, ESBLs have an extended substrate spectrum as compared with their parent types (non-ESBLs). ESBLs can also be inhibited by β-lactamase inhibitors such as clavulanic acid. Most of the known ESBLs are class A and D β-lactamases [7], but 15 class C β-lactamases with extended substrate spectra have been reported in Gram-negative pathogens isolated from clinical specimens of patients since the first description of GC1 in 1995 (Table 1). Because of the emergence and dissemination of these enzymes, we propose that these enzymes are recognized as class C ESBLs. Then class A, C, and D ESBLs would be designated aESBLs, cESBLs, and dESBLs, respectively.

**Table 1:** Epidemiology and characteristics of class C extended-spectrum β-lactamases (cESBLs)

Enzyme*	Extended Substrate Spectrum† (Parent Enzyme)	Country of Origin (Clinical Isolation)	Bacterial Species	Region (Mutation Site)‡ Causing Extended Substrate Spectrum	Reference
GC1	CAZ, ATM (P99)	Japan, 1992	*E. cloacae* GC1	Ω-loop (the insertion of Ala-Val-Arg after position 210)	[8],[10]
SRT-1	CAZ, CTX, CMX (SST-1)	Japan, 1985	*S. marcescens* GN16694	Ω-loop (Glu_213_ → Lys)	[28]
SMSA (Ser^R^)	CAZ, FEP, FPI (SLS73, Ser^S^)	France, 2000	*S. marcescens* SMSA	Ω-loop (Ser_220_ → Tyr)	[29]
CHE	CTX, FEP, FPI (P99)	France, 1998	*E. cloacae* CHE	R2-loop (a six-amino-acid-deletion, SKVALA at positions 289–294)	[11]
Ear2	CTX, FEP (Ear1)	France, 2001	*E. aerogenes* Ear2	R2-loop (Leu_293_ → Pro)	[30]
AmpC^D^	CAZ, FEP, FPI, inhibitor-sensitive (AmpC^R^, revertant)	Japan, 1994	*E. coli* HKY28	R2-loop (a tripeptide deletion, GSD, at positions 286–288)	[31]
HD	CAZ, FEP, FPI (S3)	France, 2001	*S. marcescens* HD	R2-loop (a four-amino-acid-deletion, MNGT, at positions 293–296)	[16]
EC14	CAZ, FEP (EC1)	France, 2002–2005	*E. coli* EC14	R2-loop (Val_298_ → Leu)	[17]
EC15	CAZ, FEP (EC1)	France, 2002–2005	*E. coli* EC15	R2-loop (His_296_ → Pro)	[17]
EC17	CAZ, FEP (EC1)	France, 2002–2005	*E. coli* EC17	R2-loop (His_296_ → Pro)	[17]
EC19	CAZ, FEP (EC1)	France, 2002–2005	*E. coli* EC19	R2-loop (His_296_ → Pro)	[17]
CMY-19	CAZ, FEP, FPI (CMY-9)	Japan, 1996	*K. pneuminiae* HKY327	R2-loop (Ile_292_ → Ser)	[32]
CMY-10	CAZ, IMP (P99)	Korea, 1999	*E. aerogenes* K9911729	R2-loop (a tripeptide deletion, PPA, at positions 303–305)	[24]
BER	CAZ, CTX, CRO, FEP, IMP (EC2)	France, 2006	*E. coli* BER	R2-loop (the insertion of Ala-Ala after position 293)	[18]
MHN-7.6	CAZ, FEP, FPI (MHN)	In vitro mutation	*E. coli* K12 strain MI1443	R2-loop (Val_298_ → Glu)	[9]
AmpC1	CAZ, FEP (P99)	In vitro mutation	*E. coli* JM83	R2-loop (Leu_293_ → Pro)	[33]
Seven mutant enzymes	CAZ, FEP (CMY-2)	In vitro mutation	*E. coli* DH5αE	R2-loop (Val_291_ → Ala[Gly]; Ala_292_ → Pro; Leu_293_ → Pro; Ala_294_ → Glu; Leu_296_ → Pro; Ala_298_ → Val)	[34]
520R	CAZ, FPI (S3)	In vitro mutation	*E. coli* DH5α	H-2 helix (Thr_64_ → Ile)	[35]
KL	CAZ, FEP, FPI (S4)	France, 2001	*E. coli* KL	H-11 helix (Val_350_ → Phe)	[19]

*: Crystallographic structures from distinct GC1 (Protein Data Bank [PDB] code 1GCE) and CMY-10 (PDB code 1ZKJ) only have been resolved. Ser^R^ is the in vitro site-directed mutant of SLS73 (Ser^S^). All enzymes except plasmid-encoded CMY-10 and CMY-19 are chromosomal cESBLs. All enzymes except several enzymes (Ser^R^, Ser^S^, AmpC^R^, AmpC1 [in vitro Leu-293-Pro mutant of P99], seven mutants of CMY-2, MHN-7.6, and 520R) are the naturally (clinically) occurring cESBLs produced by clinical isolates. AmpC^D^ is the only inhibitor-(tazobactam and sulbactam)sensitive cESBL.

**†:** CAZ, ceftazidime; CTX, cefotaxime; CMX, cefmenoxime; CRO, ceftriaxone; FEP, cefepime; FPI, cefpirome; IMP, imipenem; ATM, aztreonam. Each cESBL has extended its substrate specificity in comparison with each parent enzyme (non-cESBL).

**‡:** Ω-loop lays from residues 189 to 226 in P99 β-lactamase. R2-loop lays from residues 289 to 307 in CMY-10 β-lactamase. The position of the N-terminal amino acid of the mature enzyme (without the respective signal peptide) is designated as position 1 of the amino acid sequence. The tripeptide deletion of AmpC^D^ is located just before the R2-loop but causes a structural change in the R2-loop. Glu_213_ → Lys, the substitution of glutamic acid (Glu) by lysine (Lys) at residue 213.

The cESBLs were first defined as follows: i) extended specificity class C β-lactamase for GC1 in 1995 [8]; ii) extended-spectrum AmpC-type β-lactamase for MHN-7.6 in 1998 [9]; iii) extended-spectrum class C β-lactamase for GC1 in 1999 [10]; and iv) extended-spectrum AmpC β-lactamase (ESAC) for CHE in 2001 [11]. Class C β-lactamase was designated AmpC β-lactamase [12]. Therefore, extended-spectrum class C (AmpC) β-lactamase can be designated class C extended-spectrum β-lactamase (cESBL). Most cESBL (13 of 15 natural cESBLs produced by Gram-negative pathogens isolated from clinical specimens of patients: SMSA, CHE, Ear2, AmpC^D^, HD, EC14, EC15, EC17, EC19, CMY-19, BER, 520R, and KL) have extended their substrate specificity to third- and fourth-generation cephalosporins (Table 1). Some cESBLs (CMY-10 and BER) can hydrolyse carbapenems (imipenem or meropenem), which have the same substrate specificity as that of aESBLs such as GES-5 [13]. A cESBL (AmpC^D^) can be inhibited also by β-lactamase inhibitors (tazobactam and sulbactam) just like aESBLs and dESBLs. The hydrolytic efficiency (*k*_cat_/*K*_m_) of cESBLs for ceftazidime and cefotaxime was higher than or similar to that of SHV-38 [14] and CTX-M-15 [15], typical aESBLs. Some β-lactamase investigators [16]–[19] have tried to distinguish the difference between ESACs and cESBLs, but, except for cephamycins (cefoxitin and cefotetan), hydrolysis patterns do not differ between ESACs and cESBLs. Furthermore, ESBL-producing clinical isolates were also resistant to cephamycins by reduced outer membrane permeability [20]. In 2003, Hanson warned that if we have failed to distinguish between ESBL and plasmid-encoded class C β-lactamase (non-cESBL) producers, we would run the risk of the emergence of cESBLs [21]. Unfortunately, cESBLs have already emerged, and the phenotypic susceptibility testing to distinguish between aESBLs (or dESBLs) and emerging cESBLs is very difficult.

## Treatment for cESBL-Producing Gram-Negative Pathogens

The Infectious Diseases Society of America identified six top-priority dangerous pathogens (e.g., ESBL-producing *Enterobacteriaceae*, *Acinetobacter baumannii*, *Pseudomonas aeruginosa*, vancomycin-resistant *Enterococcus faecium*, methicillin-resistant *Staphylococcus aureus*, and *Aspergillus* species) for which there are few or no drugs in late-stage development, further limiting the choice of an appropriate and safe treatment for these infections [22],[23]. Three of six dangerous pathogens are antibiotic-resistant Gram-negative bacteria. Recently, antimicrobial drugs against ESBL-producing Gram-negative pathogens accounted for about 15% (2 of 13) of all antimicrobial drugs undergoing development in phase II or later clinical studies [22]. There are no drug developments against cESBL-producing Gram-negative pathogens.

Rubinstein and Zhanel, hospital physicians, have stated that physicians are increasingly forced to use the carbapenems and fluoroquinolones (ciprofloxacin or levofloxacin) as first-line therapy for ESBL-producing Gram-negative pathogens, but the situation will become even more severe as ESBL-producing organisms increasingly become concomitantly resistant to the fluoroquinolones [6]. However, we recently found that the CMY-10 cESBL had higher imipenem-hydrolysing activity than OXA-23, a class D carbapenemase [24]. Because this extended substrate spectrum of cESBLs can threaten the management of infections by Gram-negative pathogens producing these enzymes, new antimicrobial drugs against cESBL-producing Gram-negative pathogens are urgently needed. To develop these antimicrobial drugs, it is necessary to know the operative mechanism of cESBLs to extend their substrate spectrum.

## Antimicrobial Drug Development for cESBLs

**Figure 1:**
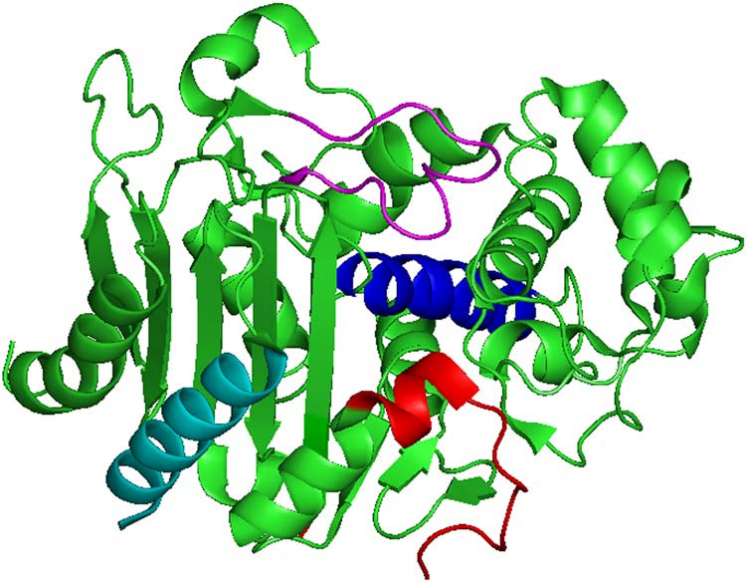
Ribbon diagram of crystallographic structure of CMY-10 (a cESBL). The image was rendered with PyMOL, available on the Internet (http://sourceforge.net/projects/pymol). The R2-loop is represented as red, while the Ω-loop, H-2 helix, and H-11 helix are depicted in violet, blue, and cyan, respectively. The R1 active site (central upper region) is surrounded by the Ω-loop and the R2 active site (central lower region) by the R2-loop and H-11 helix. The nucleophile (Ser65), attacking the carbonyl carbon of β-lactam ring, is present in the H-2 helix.

How do the cESBLs extend the substrate spectrum? The crystallographic structures can answer this question. Until now, there are two only resolved crystallographic structures of cESBLs: (i) GC1 (Protein Data Bank [PDB] code, 1GCE) [10]; and (ii) CMY-10 (PDB code, 1ZKJ) [24]. Kinetic data and the crystal structure of GC1 showed that GC1 was a natural (clinically isolated) cESBL due to the flexibility of the Ω-loop caused by the insertion of Ala-Val-Arg after position 210 [8],[10]. As shown in the Table 1, this structural characteristic of chromosomal GC1 provides insights into the molecular basis of extended substrate spectrum shown in only three cESBLs (GC1, SRT-1, and SMSA). But our kinetic data and crystal structure [24] of a plasmid-encoded cESBL (i.e., CMY-10) reveal the operative molecular strategy of most cESBLs (73%, 11 of the total 15) to extend their substrate spectrum. The region responsible for the extended substrate spectrum is the R2-loop (amino acid residues 289–307; Figure 1) [24]. Our sequence alignment of natural (clinically isolated) cESBLs shows that the R2-loop includes all regions responsible for the extended substrate spectrum in most (11 of the total 15) cESBLs: Ω-loop in three cESBLs; H-2 helix in a 520R cESBL (not natural); H-11 helix in a KL cESBL (Table 1 and Figure 1). These natural (from clinical isolates) mutations in the R2-loop can change the architecture of the active site in cESBLs, thereby affecting their hydrolysing activity. Owing to a three-amino-acid deletion (amino acid residues 303–305) in CMY-10, for example, the R2-loop in the R2 active site (i.e., the region that accommodates the R2 side-chain at C3 of the β-lactam nucleus in oxyimino-cephalosporins) displays noticeable structural alterations: the significant widening of the R2 active site. Therefore, the bulky R2 side-chain of oxyimino-cephalosporins could fit snugly into the significant widening of the R2 active site in this way. In view of no drug developments against cESBL-producing Gram-negative pathogens, new β-lactams or β-lactamase inhibitors need to be developed by the structure-based drug design (SBDD) method [25] using a similar mechanism (the significant widening of the R2 active site) of the extended substrate spectrum shown in most cESBLs. Clinically available β-lactamase inhibitors co-administered with less effective β-lactams are effective against class A β-lactamases, but show little or no activity against class C β-lactamases. Therefore, class C β-lactamases are an excellent drug target with accurate structural information [25]. Since Gram-negative pathogens producing cESBLs are increasing in emergence and spreading among organisms causing nosocomial infections (Table 1), there is an urgent need to develop an inhibitor of cESBLs or to discover new antimicrobial drugs for these cESBL-producing clinical isolates. Although large pharmaceutical companies have largely withdrawn from research and development of new antimicrobial drugs, a few academic research groups (e.g., our group, or Shoichet's laboratory [26]) and small pharmaceutical companies (e.g., Novexel [27], which has been spun out of Aventis and Anacor that has formed a worldwide strategic alliance with GlaxoSmithKline) are seeking these new β-lactamase inhibitors. The discovery of some lead compounds against CMY-10 β-lactamases by SBDD is in progress, by focused cooperation between academia and small pharmaceutical companies.

## Conclusion

Since the emergence and dissemination of fifteen class C extended-spectrum β-lactamases (ESBLs) produced by Gram-negative pathogens isolated from clinical specimens of patients, the category of ESBLs has broadened to include class C β-lactamases with extended substrate spectrum. We propose that these enzymes be recognized as class C ESBLs (cESBLs). Phenotypic susceptibility testing to distinguish the difference between organisms producing general ESBLs (e.g., aESBLs or dESBLs) or emerging cESBLs is very challenging. The difficulty in type identification of ESBLs hinders hospital infection control and the ability of the physician to prescribe the most appropriate antibiotic, thus increasing the selective pressure and generating antibiotic resistance. It is necessary for health-care professionals to recognize the presence of emerging cESBLs as a new and disturbing trend in antimicrobial resistance of Gram-negative pathogens. Furthermore, there is currently no drug development in progress against cESBL-producing Gram-negative pathogens. Therefore, there is a tremendous need for the development of new β-lactams or β-lactamase inhibitors by the structure-based drug-design method using the similar structural mechanism (the significant widening of the R2 active site) of the extended substrate spectrum shown in most cESBLs.

## Accession Number

The Protein Data Bank (PDB, http://www.rcsb.org/pdb/) accession code for the protein discussed in this paper is CMY-10 (1ZKJ, [24]).
